# High-Intensity Exercise With Blood Flow Restriction or in Hypoxia as Valuable Spaceflight Countermeasures?

**DOI:** 10.3389/fphys.2019.01266

**Published:** 2019-10-02

**Authors:** Sarah J. Willis, Fabio Borrani, Grégoire P. Millet

**Affiliations:** Faculty of Biology and Medicine, Institute of Sport Sciences, University of Lausanne, Lausanne, Switzerland

**Keywords:** BFR, altitude, vascular, perfusion, blood volume, occlusion

## Introduction

Determining effective countermeasures to the physical issues of microgravity, radiation, and isolation (i.e., decreased aerobic capacity and cardiovascular dysfunction, along with muscle atrophy, bone and muscle loss, and hypocapnia) is crucial for human space flights (Demontis et al., 2017). Since the initial Apollo Program (1961–1972), there is still a great challenge in determining and replicating the most efficient exercise countermeasures mainly due to restricted time, habitable volume, and maintenance of the life support systems (Scott et al., 2019). Additionally, risks of pressure loss in the space habitat and thus compression sickness together with a hypoxic environment must be considered and should be combated through acclimatization and sufficient training/preparation (Lewis, 2018; Millet, in press). When considering time restrictions for spaceflight preparation, in-flight exercise time, and access for exercise, combining exercise methods for rapid adaptation appears paramount.

The hypoxic stimulus is an important consideration for spaceflight (Bodkin et al., 2006) and requires an effective pre-acclimatization. A balance is needed between the barometric conditions of the habitat and the safety threshold of oxygen concentrations due to flammability. In this context, it is applicable to incorporate hypoxic training methods for acclimation with preference of hypobaric hypoxia (decreased barometric pressure and oxygen fraction) over normobaric hypoxia due to the risks associated with excessive oxygen in the atmosphere of the space habitat (Millet, in press). It has been shown that passive hypoxic exposure leads to negligible adaptations in skeletal muscle tissue (Lundby et al., 2009), while the combination of prolonged passive exposure to hypoxia with exercise, specifically high-intensity exercise (Live High-Train Low and High, LHTLH) induces beneficial transcriptional responses, which are not present with passive exposure only (i.e., traditional “Live High-Train Low” training method) (Brocherie et al., 2018). Among the specific responses associated to LHTLH, there are increased mRNA levels in the vastus lateralis involved in oxygen signaling, oxygen carrier, mitochondrial biogenesis, and metabolism (Brocherie et al., 2018), as well as positive functional adaptations as shown by the improved oxidative capacity in type I and type II fibers, while maintaining fiber size (van der Zwaard et al., 2018). During acute hypoxic exposure, the lowered oxygen delivery induces a lower aerobic performance (Lundby et al., 2009; Slivka, 2017). Though exercise helps to protect skeletal muscle from these declines, there is an upregulation of the VEGF and glycolytic genes (amongst hundreds of others) through the HIF-1 oxygen-sensing pathway. Moreover, chronic exposure to hypoxia leads to improved mitochondrial and capillary density and enhanced oxidative capacity (Lundby et al., 2009; Slivka, 2017). Altogether, the combinations of passive hypoxic exposure as experienced during space flights and high-intensity exercise in hypoxia appear as adequate strategies for improving peak power production and maximal oxygen consumption.

## High-intensity Exercise With Systemic Hypoxia

The effect of combining high-intensity exercise with systemic hypoxia elicits greater muscle perfusion and oxygenation (Brocherie et al., 2017) along with enhanced muscle transcriptional responses of the vastus lateralis when compared to normoxia (Faiss et al., 2013; Brocherie et al., 2018). Specifically, these researchers identified molecular adaptations which improve oxygen signaling (HIF-1α), oxygen carrying (Mb), and pH regulation (CA3) via upregulation of genes, along with downregulation of genes involved in mitochondrial biogenesis (TFAM and PGC-1α). These alterations suggest improved anaerobic glycolytic activity of the muscle (Faiss et al., 2013) and improved fast-twitch fiber recruitment in the vastus lateralis after repeated sprint training in hypoxia (McDonough et al., 2005), particularly by compensatory vasodilation and increased rate of phosphocreatine resynthesis (Hoppeler and Vogt, 2001; Zoll et al., 2006) which are linked to performance enhancement. It is important to consider that not all muscles react in similar ways regarding growth, atrophy, and their response to hypoxic conditions, as they are highly influenced by changes in perfusion pressure (Fitzpatrick et al., 1996). As such, any decrease in perfusion pressure across a physiological range to the contracting muscles results in an increase in muscle activation to maintain a constant force output (Fitzpatrick et al., 1996). Furthermore, vascular adaptations occur after maximal intensity training in hypoxia through increased changes in blood perfusion (via changes in total hemoglobin) and contribute to the delay of fatigue in both the lower (Faiss et al., 2013) and upper body (Faiss et al., 2014) (vastus lateralis and triceps brachii, respectively). During high-intensity exercise in hypoxia, there are great stresses placed on oxygen transport and the vascular system. Due to the reduced oxygen availability in the environment, vasodilation occurs to increase blood flow to the muscle tissue and maintain oxygen delivery. Additionally, the increased changes in blood perfusion due to the combination of high-intensity and hypoxic stress may be a stimulus for altering vascular blood flow regulation due to neural, metabolic, and mechanical influences. Of interest is that short blocks (i.e., as little as 4–8 repeated sprint training sessions in hypoxia), led to improved performance for elite athletes in different sports as cycling (Faiss et al., 2013), cross-country skiing (Faiss et al., 2014), rugby (Beard et al., 2018, 2019), and tennis (Brechbuhl et al., 2018). To our knowledge, there is no data available on the effects of such exercises in astronauts and other participants during spaceflights but one may speculate that performing high-intensity exercise training in hypoxia may be a valuable and practical method for exercise countermeasure during spaceflight missions.

## Blood Flow Restriction

Other methods exist for inducing a hypoxic stimulus to muscles: The compression of the vasculature proximal to the skeletal muscle results in inadequate oxygen supply (hypoxia) within the muscle tissue (Patterson et al., 2019). Rather than reducing the oxygen in the atmospheric environment on a systemic level, hypoxia can occur on a local level when an external pressure is applied to the limbs to create partial restriction of blood flow (blood flow restriction, BFR). In this manner, the vascular occlusion (or ischemia) diminishes blood flow by vascular resistance and venous return is substantially limited (Kaijser et al., 1990). BFR has been shown to upregulate the mRNA expression in the vastus lateralis of the vascular endothelial growth factor (VEGF and VEGFR-2) along with HIF-1α and eNOS, suggesting angiogenesis due to the increased stimuli of ischemic and shear stress during low-load resistance exercise (Scott et al., 2014; Taylor et al., 2016; Ferguson et al., 2018). The AMPK pathway is responsible for regulating energy metabolism where kinases are activated in response to stresses that deplete ATP including those of hypoxia and ischemia. This pathway activates catabolism while suppressing synthesis, leading to imbalanced energy metabolism. Further, reactive oxygen species (ROS) production is increased in these conditions due to high levels of metabolic stress and leads to an unbalanced oxidative status. Furthermore, the lower partial pressure of oxygen during BFR exercise limits the amount of ROS production at least acutely, lowering the mitochondrial H_2_O_2_ emission rates and electron leak to ROS (Petrick et al., 2019). The knowledge of the mechanisms and adaptations are limited during continuous exercise training with BFR. With low-intensity exercise of about 40% VO_2max_, BFR has shown to increase strength and hypertrophy (Slysz et al., 2016; Conceicao et al., 2019), in both walking (Abe et al., 2006) and leg-cycling exercise (Abe et al., 2010; Conceicao et al., 2019) in as early as 3 weeks (Abe et al., 2006) and with greatest effectiveness after ≥6 weeks of training (Slysz et al., 2016). Furthermore, BFR training during low-intensity walking and leg-cycling has been shown to increase strength as well as aerobic capacity in young (Slysz et al., 2016), old (Abe et al., 2010), and trained participants (Park et al., 2010). Moreover, the use of BFR techniques are important to consider as a method to counteract muscle degeneration and sarcopenia over a range of passive, resistance training, and continuous exercise protocols (Patterson et al., 2019). BFR provides a potent, gravitational-like stimulus on the cardiovascular system and may counteract the orthostatic intolerance upon return to Earth (Iida et al., 2007; Nakajima et al., 2008). Research has demonstrated that BFR was similar regarding physiological and hemodynamic responses to lower body negative pressure (LBNP) eliciting blood pooling in the lower limbs, reduced venous return, and hemodynamic responses of decreased stroke volume, increased cardiac output, and increased total peripheral resistance as similar to LBNP (Stevens and Lamb, 1965; Tomaselli et al., 1987; Lathers and Charles, 1993). Researchers suggested that the use of bilateral thigh BFR likely partially simulates the hemodynamic, systemic cardiovascular, autonomic nervous, and hormonal effects of orthostasis as seen during simulated weightlessness (Nakajima et al., 2008). Additionally suggesting that BFR training may provide an appropriate countermeasure to combat the associated declines in atrophy associated with weightlessness (Nakajima et al., 2008). Further, the altered hemodynamic and chemical/metabolic signals during BFR exercise (Ferguson et al., 2018) likely effect the improvement in vascular function through remodeling of the arterial lumen, which contributes to the cardio-protective effects of exercise (Thijssen et al., 2012). Additionally, the magnitude and location (conduit, resistance, capillary vessel) of the vascular adaptations depend on the intensity, volume of exposure, and mode of training (Green et al., 2011). Indeed, when combining BFR and/or systemic hypoxia with high-intensity exercise, a robust stimulus is placed on the vascular mechanisms (Willis et al., 2018, 2019a,b). These different methods of systemic and local hypoxia along with the differing underlying mechanisms (metabolic vasodilation and vascular resistance, respectively) can provide a great stimulus alone or in combination to alter vascular conductance and blood flow regulation.

## High-intensity Exercise With Blood Flow Restriction

In general, high-intensity exercise reduces tissue oxygen availability and therefore increases the oxygen extraction in order to maintain oxygen delivery (Granger and Shepherd, 1973). This is further challenged when high-intensity exercise is combined with hypoxia (Casey and Joyner, 2012). When high-intensity exercise is performed with BFR, in addition to a strong deoxygenation due to the localized hypoxia, there is a larger increase in the changes in blood perfusion in both legs and arms (vastus lateralis and biceps brachii, respectively) (Willis et al., 2018, 2019b). High-intensity exercise with BFR is able to create a potent stimulus via vascular resistance and altered vasodilatory responses, and was shown to be more robust than with systemic hypoxia in both legs and arms (Peyrard et al., 2019; Willis et al., 2019a,b). In fact, during high-intensity exercise with a certain level of BFR, an additional stimulus of systemic hypoxia is likely blunted (Willis et al., 2019a). At this moment, the mechanisms of the interaction between hypertrophic, hypoxic, and vascular adaptations remain elusive. Though studies have not yet been conducted in a microgravity environment, incorporating these methods of hypoxia and BFR during high-intensity exercise elicits responses that are likely beneficial to improve the physical capacities, since deconditioning and muscle atrophy, are present during spaceflight missions.

## Practical Applications to Spaceflight Participants

Incorporating high-intensity exercise training in hypoxia with legs or arms is beneficial for improving performance and delaying fatigue by way of adaptations to improve muscle oxygenation, specifically of the vastus lateralis and triceps brachii (Faiss et al., 2013, 2014). Furthermore, acutely performing high-intensity exercise with BFR elicits greater reactivity regarding changes in blood volume and oxygenation than with systemic hypoxia alone in both the legs and arms (vastus lateralis and biceps brachii, respectively) (Willis et al., 2018, 2019b), with a greater effect in the arms (Willis et al., 2019a). Altogether, utilizing these training methods is beneficial for increasing the efficiency of blood and oxygen transport allowing increased vascular proficiency. The implementation of short training blocks can be valuable to induce a rapid and robust stimulus in a short time frame, thus providing a time-saving strategy and effective method for adaptations to occur. While the optimal stimulus depends on the population of interest, suggested training recommendations are as follows. The high-intensity exercise protocol should last ~60 min including the warm-up and cool-down periods and be performed 2–3 times a week during blocks of training (2–5 weeks duration) as part of a periodized training program. During each session, the athlete performs a series of 3–4 sets of 4–7 maximal to supra-maximal “all-out” sprints (4–15s duration) in hypoxia (3,000–3,800 m and 14.2–12.8% FiO_2_) and/or with BFR of about 45% of total occlusion pressure. It is important to achieve a specific sprint-to-rest ratio of 1:2–1:4 with inter-set recovery about 3–5 min without occlusion. This training has been successful in inducing adaptations in hypoxic conditions as shown by many researchers (Brocherie et al., 2017). This specific training should be performed in alteration with low-intensity exercise to maintain and develop basic fitness along with aerobic metabolism.

## Conclusion

Performing high-intensity exercise with BFR or hypoxia are promising training methods for both legs and arms in order to increase physical conditions prior to, during, and after returning from spaceflight missions. This training may rapidly induce vascular adaptations and allow for combined metabolic and hypertrophic effects to counteract the decreased aerobic capacity and muscle atrophy occurring with microgravity, and allow adaptations to occur which may enhance endothelial function and lead to improved tissue oxygenation. Altogether, high-intensity exercise with BFR or in the hypoxic condition of the space vehicle should be considered as a practical, efficient, time effective, and influential countermeasure for space travelers.

## Author Contributions

All authors listed have made a substantial, direct and intellectual contribution to the work, and approved it for publication.

### Conflict of Interest

The authors declare that the research was conducted in the absence of any commercial or financial relationships that could be construed as a potential conflict of interest.

## References

[B1] Abe T. Fujita S. Nakajima T. Sakamaki M. Ozaki H. Ogasawara R.. (2010). Effects of low-intensity cycle training with restricted leg blood flow on thigh muscle volume and VO2MAX in young men. J. Sports Sci. Med. 9, 452–458. 24149640 PMC3761718

[B2] Abe T. Kearns C. F. Sato Y. (2006). Muscle size and strength are increased following walk training with restricted venous blood flow from the leg muscle, Kaatsu-walk training. J. Appl. Physiol. 100, 1460–1466. 10.1152/japplphysiol.01267.2005 16339340

[B3] Beard A. Ashby J. Chambers R. Brocherie F. Millet G. P. (2018). Repeated-sprint training in hypoxia in international rugby union players. Int. J. Sports Physiol. Perform. 14, 850–854. 10.1123/ijspp.2018-0170 30569787

[B4] Beard A. Ashby J. Kilgallon M. Brocherie F. Millet G. P. (2019). Upper-body repeated-sprint training in hypoxia in international rugby union players. Eur. J. Sport Sci. 19, 1175-1183. 10.1080/17461391.2019.1587521 30880627

[B5] Bodkin D. Escalera P. Bocam K. (2006). A human lunar surface base and infrastructure solution, in AS-10: Space Architecture Symposium: Lunar and Planetary Surface Systems and Construction I (San Jose, CA: AIAA), 1–17. 10.2514/6.2006-7336

[B6] Brechbuhl C. Brocherie F. Millet G. P. Schmitt L. (2018). Effects of repeated-sprint training in hypoxia on tennis-specific performance in well-trained players. Sports Med. Int. Open 2, E123–E132. 10.1055/a-0719-4797 30539129 PMC6259464

[B7] Brocherie F. Girard O. Faiss R. Millet G. P. (2017). Effects of repeated-sprint training in hypoxia on sea-level performance: a meta-analysis. Sports Med. 47, 1651–1660. 10.1007/s40279-017-0685-3 28194720

[B8] Brocherie F. Millet G. P. D'Hulst G. Van Thienen R. Deldicque L. Girard O. (2018). Repeated maximal-intensity hypoxic exercise superimposed to hypoxic residence boosts skeletal muscle transcriptional responses in elite team-sport athletes. Acta Physiol. 222:e12851. 10.1111/apha.12851 28103427

[B9] Casey D. P. Joyner M. J. (2012). Compensatory vasodilatation during hypoxic exercise: mechanisms responsible for matching oxygen supply to demand. J. Physiol. 590, 6321–6326. 10.1113/jphysiol.2012.242396 22988134 PMC3533194

[B10] Conceicao M. S. Junior E. M. M. Telles G. D. Libardi C. A. Castro A. Andrade A. L. L.. (2019). Augmented anabolic responses after 8-wk cycling with blood flow restriction. Med. Sci. Sports Exerc. 51, 84–93. 10.1249/MSS.0000000000001755 30113523

[B11] Demontis G. C. Germani M. M. Caiani E. G. Barravecchia I. Passino C. Angeloni D. (2017). Human pathophysiological adaptations to the space environment. Front. Physiol. 8:547. 10.3389/fphys.2017.00547 28824446 PMC5539130

[B12] Faiss R. Leger B. Vesin J. M. Fournier P. E. Eggel Y. Deriaz O.. (2013). Significant molecular and systemic adaptations after repeated sprint training in hypoxia. PLoS ONE 8:e56522. 10.1371/journal.pone.0056522 23437154 PMC3577885

[B13] Faiss R. Willis S. Born D. P. Sperlich B. Vesin J. M. Holmberg H. C.. (2014). Repeated double-poling sprint training in hypoxia by competitive cross-country skiers. Med. Sci. Sports Exerc. 47, 809–817. 10.1249/MSS.0000000000000464 25083727

[B14] Ferguson R. A. Hunt J. E. A. Lewis M. P. Martin N. R. W. Player D. J. Stangier C.. (2018). The acute angiogenic signalling response to low-load resistance exercise with blood flow restriction. Eur. J. Sport Sci. 18, 397–406. 10.1080/17461391.2017.1422281 29343183

[B15] Fitzpatrick R. Taylor J. L. McCloskey D. I. (1996). Effects of arterial perfusion pressure on force production in working human hand muscles. J. Physiol. 495 (Pt 3), 885–891. 10.1113/jphysiol.1996.sp021640 8887790 PMC1160789

[B16] Granger H. J. Shepherd A. P. Jr. (1973). Intrinsic microvascular control of tissue oxygen delivery. Microvasc Res 5, 49–72. 10.1016/S0026-2862(73)80006-8 4684756

[B17] Green D. J. Spence A. Halliwill J. R. Cable N. T. Thijssen D. H. (2011). Exercise and vascular adaptation in asymptomatic humans. Exp. Physiol. 96, 57–70. 10.1113/expphysiol.2009.048694 20971800

[B18] Hoppeler H. Vogt M. (2001). Muscle tissue adaptations to hypoxia. J. Exp. Biol. 204(Pt 18), 3133–3139. 11581327 10.1242/jeb.204.18.3133

[B19] Iida H. Kurano M. Takano H. Kubota N. Morita T. Meguro K.. (2007). Hemodynamic and neurohumoral responses to the restriction of femoral blood flow by KAATSU in healthy subjects. Eur. J. Appl. Physiol. 100, 275–285. 10.1007/s00421-007-0430-y 17342543

[B20] Kaijser L. Sundberg C. J. Eiken O. Nygren A. Esbjornsson M. Sylven C.. (1990). Muscle oxidative capacity and work performance after training under local leg ischemia. J. Appl. Physiol. 69, 785–787. 10.1152/jappl.1990.69.2.785 2146245

[B21] Lathers C. M. Charles J. B. (1993). Use of lower body negative pressure to counter symptoms of orthostatic intolerance in patients, bed rest subjects, and astronauts. J. Clin. Pharmacol. 33, 1071–1085. 10.1002/j.1552-4604.1993.tb01944.x 8300890

[B22] Lewis P. (2018). Is hypoxia-induced skeletal muscle dysfunction lost in space or just a matter of a time? J. Physiol. 596, 2959–2960. 10.1113/JP276033 29509275 PMC6068208

[B23] Lundby C. Calbet J. A. Robach P. (2009). The response of human skeletal muscle tissue to hypoxia. Cell Mol. Life Sci. 66, 3615–3623. 10.1007/s00018-009-0146-8 19756383 PMC11115669

[B24] McDonough P. Behnke B. J. Padilla D. J. Musch T. I. Poole D. C. (2005). Control of microvascular oxygen pressures in rat muscles comprised of different fibre types. J. Physiol. 563(Pt 3), 903–913. 10.1113/jphysiol.2004.079533 15637098 PMC1665627

[B25] Millet G. P. (in press). Hypobaric hypoxia as part of the equation. N Engl J Med.

[B26] Nakajima T. Iida H. Kurano M. Takano H. Morita T. Meguro K.. (2008). Hemodynamic responses to simulated weightlessness of 24-h head-down bed rest and KAATSU blood flow restriction. Eur. J. Appl. Physiol. 104, 727–737. 10.1007/s00421-008-0834-3 18651162

[B27] Park S. Kim J. K. Choi H. M. Kim H. G. Beekley M. D. Nho H. (2010). Increase in maximal oxygen uptake following 2-week walk training with blood flow occlusion in athletes. Eur. J. Appl. Physiol. 109, 591–600. 10.1007/s00421-010-1377-y 20544348

[B28] Patterson S. D. Hughes L. Warmington S. Burr J. Scott B. R. Owens J.. (2019). Blood flow restriction exercise position stand: considerations of methodology, application, and safety. Front. Physiol. 10:533. 10.3389/fphys.2019.00533 31156448 PMC6530612

[B29] Petrick H. L. Pignanelli C. Barbeau P. A. Churchward-Venne T. A. Dennis K. M. J. J. van Loon L. J. C. (2019). Blood flow restricted resistance exercise and reductions in oxygen tension attenuate mitochondrial H2O2 emission rates in human skeletal muscle. J. Physiol. 597, 3985–3997. 10.1113/JP277765. 31194254

[B30] Peyrard A. Willis S. J. Place N. Millet G. P. Borrani F. Rupp T. (2019). Neuromuscular evaluation of arm-cycling repeated sprints under hypoxia and/or blood flow restriction. Eur. J. Appl. Physiol. 119, 1533–1545. 10.1007/s00421-019-04143-4 31011807

[B31] Scott B. R. Slattery K. M. Sculley D. V. Dascombe B. J. (2014). Hypoxia and resistance exercise: a comparison of localized and systemic methods. Sports Med. 44, 1037–1054. 10.1007/s40279-014-0177-7 24715613

[B32] Scott J. P. R. Weber T. Green D. A. (2019). Introduction to the frontiers research topic: optimization of exercise countermeasures for human space flight - lessons from terrestrial physiology and operational considerations. Front. Physiol. 10:173. 10.3389/fphys.2019.00173 30899226 PMC6416179

[B33] Slivka D. R. (2017). Skeletal muscle response to hypoxia. Acta Physiol. 220, 9–10. 10.1111/apha.12797 27589659

[B34] Slysz J. Stultz J. Burr J. F. (2016). The efficacy of blood flow restricted exercise: a systematic review & meta-analysis. J. Sci. Med. Sport. 19, 669–675. 10.1016/j.jsams.2015.09.005 26463594

[B35] Stevens P. M. Lamb L. E. (1965). Effects of lower body negative pressure on the cardiovascular system. Am. J. Cardiol. 16, 506–515. 10.1016/0002-9149(65)90027-5 5319567

[B36] Taylor C. W. Ingham S. A. Ferguson R. A. (2016). Acute and chronic effect of sprint interval training combined with postexercise blood-flow restriction in trained individuals. Exp. Physiol. 101, 143–154. 10.1113/EP085293 26391312

[B37] Thijssen D. H. Cable N. T. Green D. J. (2012). Impact of exercise training on arterial wall thickness in humans. Clin. Sci. 122, 311–322. 10.1042/CS20110469 22150253 PMC3233305

[B38] Tomaselli C. M. Frey M. A. Kenney R. A. Hoffler G. W. (1987). Hysteresis in response to descending and ascending lower-body negative pressure. J. Appl. Physiol. 63, 719–725. 10.1152/jappl.1987.63.2.719 3654433

[B39] van der Zwaard S. Brocherie F. Kom B. L. G. Millet G. P. Deldicque L. van der Laarse W. J.. (2018). Adaptations in muscle oxidative capacity, fiber size, and oxygen supply capacity after repeated-sprint training in hypoxia combined with chronic hypoxic exposure. J. Appl. Physiol. 124, 1403–1412. 10.1152/japplphysiol.00946.2017 29420150

[B40] Willis S. J. Alvarez L. Borrani F. Millet G. P. (2018). Oxygenation time course and neuromuscular fatigue during repeated cycling sprints with bilateral blood flow restriction. Physiol. Rep. 6:e13872. 10.14814/phy2.13872 30295004 PMC6174122

[B41] Willis S. J. Borrani F. Millet G. P. (2019a). Leg- vs arm-cycling repeated sprints with blood flow restriction and systemic hypoxia. Eur. J. Appl. Physiol. 119, 1819–1828. 10.1007/s00421-019-04171-0 31187281

[B42] Willis S. J. Peyrard A. Rupp T. Borrani F. Millet G. P. (2019b). Vascular and oxygenation responses of local ischemia and systemic hypoxia during arm cycling repeated sprints. J. Sci. Med. Sport. 22, 1151–1156. 10.1016/j.jsams.2019.05.001 31104973

[B43] Zoll J. Ponsot E. Dufour S. Doutreleau S. Ventura-Clapier R. Vogt M.. (2006). Exercise training in normobaric hypoxia in endurance runners. III. Muscular adjustments of selected gene transcripts. J. Appl. Physiol. 100, 1258–1266. 10.1152/japplphysiol.00359.2005 16540710

